# Effectiveness of late gadolinium enhancement to improve outcomes prediction in patients referred for cardiovascular magnetic resonance after echocardiography

**DOI:** 10.1186/1532-429X-15-6

**Published:** 2013-01-16

**Authors:** Timothy C Wong, Kayla Piehler, Kathy S Puntil, Diego Moguillansky, Christopher G Meier, Joan M Lacomis, Peter Kellman, Stephen C Cook, David S Schwartzman, Marc A Simon, Suresh R Mulukutla, Erik B Schelbert

**Affiliations:** 1 Department of Medicine, University of Pittsburgh School of Medicine, Pittsburgh, PA; 2 Cardiovascular Magnetic Resonance Center, UPMC, Pittsburgh, PA; 3 Heart and Vascular Institute, UPMC, Pittsburgh, PA; 4 Department of Radiology, University of Pittsburgh School of Medicine, Pittsburgh, PA; 5 National Heart, Lung, Blood Institute, Bethesda, MD, Maryland; 6 Clinical and Translational Science Institute, University of Pittsburgh, Pittsburgh, PA; 7 Department of Bioengineering, University of Pittsburgh, Pittsburgh, PA; 8 Center for Quality, Outcomes, and Clinical Research, UPMC, Pittsburgh, PA

**Keywords:** Echocardiography, Cardiovascular magnetic resonance imaging, Late gadolinium enhancement, Ejection fraction, Comparative effectiveness

## Abstract

**Background:**

Echocardiography (echo) is a first line test to assess cardiac structure and function. It is not known if cardiovascular magnetic resonance (CMR) with late gadolinium enhancement (LGE) ordered during *routine* clinical practice in selected patients can add additional prognostic information after *routine* echo. We assessed whether CMR improves outcomes prediction after contemporaneous echo, which may have implications for efforts to optimize processes of care, assess effectiveness, and allocate limited health care resources.

**Methods and results:**

We prospectively enrolled 1044 consecutive patients referred for CMR. There were 38 deaths and 3 cardiac transplants over a median follow-up of 1.0 years (IQR 0.4-1.5). We first reproduced previous survival curve strata (presence of LGE and ejection fraction (EF) < 50%) for transplant free survival, to support generalizability of any findings. Then, in a subset (n = 444) with contemporaneous echo (median 3 days apart, IQR 1–9), EF by echo (assessed visually) or CMR were modestly correlated (R^2^ = 0.66, p < 0.001), and 30 deaths and 3 transplants occurred over a median follow-up of 0.83 years (IQR 0.29-1.40). CMR EF predicted mortality better than echo EF in univariable Cox models (Integrated Discrimination Improvement (IDI) 0.018, 95% CI 0.008-0.034; Net Reclassification Improvement (NRI) 0.51, 95% CI 0.11-0.85). Finally, LGE further improved prediction beyond EF as determined by hazard ratios, NRI, and IDI in all Cox models predicting mortality or transplant free survival, adjusting for age, gender, wall motion, and EF.

**Conclusions:**

Among those referred for CMR after echocardiography, CMR with LGE further improves risk stratification of individuals at risk for death or death/cardiac transplant.

## Background

In routine clinical practice, it is not known whether cardiovascular magnetic resonance (CMR) with left ventricular ejection fraction (EF) measurement and tissue characterization with late gadolinium enhancement (LGE) can add any additional prognostic information after 2-dimensional (2D) echocardiography. A clinical question frequently posed to the CMR practitioner is the following: “We already have an echocardiogram on this patient; why do we need CMR?” Indeed, echocardiography remains a first line test for evaluating patients with known or suspected cardiac disease. Both echocardiography and CMR measure EF, a principal determinant of risk that often governs medical, surgical, and device-based therapies. CMR measures EF with 3-dimensional (3D) techniques which appear superior to 2D techniques employed by echocardiography [1] that are prone to error related to suboptimal acoustic windows, off axis images, foreshortening of the apex, endocardial dropout, and confounding by wall motion abnormalities. [2,3] In addition, while echocardiography detects prognostically relevant wall motion abnormalities, [4] it cannot readily characterize myocardial tissue composition. Myocardial tissue composition characterized by late gadolinium enhancement (LGE), may improve outcomes prediction beyond EF. [5,6] Whether the potential advantages of CMR regarding EF and LGE translate into added prognostic ability after 2D echocardiography in selected patient populations remains unproven.

Demonstrating prognostic contributions for a given modality has considerable relevance from several perspectives. Both patients and their physicians rely on risk stratification to individualize care and optimize therapy, and there is a need to understand the strengths and limitations of imaging modalities. Comparisons of risk assessment across modalities underlie efforts to assess comparative effectiveness, optimize processes of care, and allocate limited healthcare resources. If CMR with LGE further refines risk stratification after echocardiography in selected clinical populations, then CMR would demonstrate a potential to improve care through improved patient selection for interventions and more efficient allocation of healthcare resources, despite the higher expense of CMR upfront.

We studied only patients referred for clinical CMR when deemed appropriate by their physicians. The specific aim was to assess in a “real world” population the *effectiveness* of CMR after echocardiography for the prediction of 1) all-cause mortality, or 2) cardiac transplant-free survival. Clinicians used these data for patient management, and so they are inherently valid and legitimate parameters worthy of further study. This study was not designed to compare *efficacy* which has been previously reported. [1,7,8] Thus, in this effectiveness study we prospectively enrolled consecutive patients at the time of clinical CMR referral at our institution and compiled echocardiography and CMR data and “aged with our cohort”. [9] The *a priori* intent of CMR and echocardiography data collection at time of CMR was to compare their ability to predict subsequent outcomes. First, we sought to verify generalizability and confirm the findings of previous work demonstrating that LGE adds incremental prognostic value beyond EF by CMR in consecutive patients. [5] Demonstrating generalizability, i.e., that CMR data are reproducible across CMR centers, is useful to support any subsequent claims of effectiveness. Then, in a subset with contemporaneous echocardiography we tested the hypothesis that EF assessed by CMR was a stronger predictor than EF assessed qualitatively. Finally, we tested the hypothesis that LGE would provide added predictive ability beyond contemporaneous echocardiography EF as determined by net reclassification improvement (NRI) and integrated discrimination improvement (IDI) from Cox regression models predicting either death or transplant free survival. Together, these data would support the potential for added prognostic value of CMR after echocardiography in selected patients.

## Methods

### Patient population

After approval of the protocol from the Institutional Review Board (IRB) which complied with the Declaration of Helsinki, we prospectively recruited 1,044 consecutive adult patients as they were referred for clinical CMR at the University of Pittsburgh Medical Center (UPMC) Cardiovascular Magnetic Resonance Center. Inclusion criteria were the provision of verbal and written informed consent, the ability to undergo a complete contrast enhanced CMR scan which required a glomerular filtration rate ≥30 mL/min/1.7 m^2^ and the absence of other known contraindications to CMR scanning. This group constituted the *“reproducibility cohort”* to assess the reproducibility of the general findings of Cheong et al. [5].

In the sample, there was a subset of 451 individuals who had contemporaneous echocardiography defined arbitrarily as within 3 weeks of CMR (median 3 days, IQR 1–9 days). Patients with known or suspected stress-induced (“takotsubo”) cardiomyopathy (n = 6), a condition where the EF can change rapidly, were excluded from the cohort. Those with significantly large acute myocardial infarctions or areas at risk, defined arbitrarily but conservatively by peak troponin I levels >10 ng/mL (0.1 ng/ml detection limit), were excluded (n = 1) if the CMR scan was acquired more than 6 days apart, due to concerns of confounding related to interval recovery of stunned myocardium. Thus, 444 patients formed the *“echocardiography cohort.”* CMR and echocardiograms were acquired between September 2009 to August 2011.

We captured data elements that reflect routine clinical practice in order to maximize generalizability. Demographic and comorbidity data were determined according to the medical record and patient interview at the time of CMR scanning. Data related to CMR or echocardiography were recorded from the final clinical reports. Study data were collected and managed using REDCap (Research Electronic Data Capture) electronic data capture tools hosted at the University of Pittsburgh. [10] Vital status was ascertained by Social Security Death Index queries and medical record review. To identify individuals who received cardiac transplant, we cross referenced our database with the University of Pittsburgh Medical Center Cardiothoracic Transplantation Program’s Transplant Patient Management System, which prospectively collects data for all transplant patients and is IRB approved.

### Echocardiography

All echocardiography exams except for 27 in the echocardiography cohort and 129 in the reproducibility cohort were acquired at the University of Pittsburgh Medical Center with commercially available systems read by 9 experienced echocardiographers. This echocardiography laboratory is accredited by the Inter Societal Accreditation Commission - Echocardiography (ICAEL). Nearly all EFs were assessed by visual estimation which assigned values with discrete increments of ∆ 5% from 0-75% with the exception of 7 subjects who had EF measured by biplane Simpsons’ method. [2] Echocardiography contrast agents to improve endocardial border definition were used in 37 subjects. The presence of regional wall motion abnormalities which may be viewed as a potential surrogate for myocardial infarction or LGE was also recorded.

### CMR scans

#### Cine CMR

All patients underwent clinical CMR scans by dedicated CMR technologists with a 1.5T Siemens Magnetom Espree (Siemens Medical Solutions, Erlangen, Germany) and a 32 channel phased array cardiovascular coil. The exam included standard breath held segmented cine imaging with steady state free precession (SSFP). [11] Left ventricular EF was measured without geometric assumptions from short axis stacks of end diastolic and end systolic cine frames (slices 6 mm thick, 4 mm apart, 30 frames per cardiac cycle). [11] In the presence of arrhythmia or inability to breath hold (n = 38), we employed non-segmented real-time cines over at least 4 seconds to identify end systole accurately with parallel imaging factors of 4, and lower spatial resolution (e.g., 96×192 matrix) to maximize temporal resolution (e.g., <75 msec/frame) without compromising the ability to ascertain myocardial borders.

#### Late gadolinium enhancement

Late gadolinium enhancement (LGE) imaging [11] was performed 10–20 minutes after a 0.2 mmol/kg intravenous gadoteridol bolus (Prohance, Bracco Diagnostics, Princeton, NJ). To optimize detection of grossly evident LGE abnormality, we used phase sensitive inversion recovery pulse sequences to increase signal to noise ratios, correct for surface coil intensity variation, and render signal intensity proportional to T1 recovery; we used both segmented gradient echo and single shot steady state precession sequences [12,13].

### Statistical analysis

Categorical variables were summarized as percentages, and continuous variables were summarized by their median and interquartile range. Nonparametric statistics were employed for continuous variables after non-normality was identified by visual inspection of distributions with further confirmation by the Shapiro Wilk test. Since echocardiography EFs were reported as small ranges of ∆ 5%, we used the midpoint of the range to compare to CMR EFs which were discrete values. Statistical tests were two sided, and p < 0.05 was considered significant. Chi square tests compared associations between categorical variables, and Wilcoxon rank sum tests compared associations between continuous variables. Linear regression and Bland-Altman analysis were performed to compare contemporaneous EF derived from echocardiography or CMR. Survival analysis employed the log rank test or Cox regression. Results were similar regardless of whether we used the date of CMR as time zero for follow-up or the date of echo as time zero for follow-up; we report the latter since CMR mostly followed echocardiography. The number of events limited the number of predictor variables to permit roughly 10 events per predictor variable. Proportional hazards assumptions were verified by Schoenfeld residuals and nonsignificant time interaction terms for EF and LGE. There was no statistical interaction in Cox regression models between LGE and EF regardless of EF modality or endpoint (death or transplant free survival). We summarized the added predictive ability of CMR data by computing the integrated discrimination improvement (IDI) and net reclassification improvement (NRI). The IDI measures the improvement of a new model’s average sensitivity without sacrificing average specificity. The NRI measures the correctness of reclassification of subjects based on their predicted probabilities of events using the new model [14,15]:

(1) $$\begin{array}{l} \mathrm{NRI}=\left(\#\mathrm{events}\;\mathrm{moving}\;\mathrm{up}-\#\mathrm{events}\;\mathrm{moving}\;\mathrm{down}\right)\\ \;/\#\mathrm{events})+\left(\#\mathrm{nonevents}\;\mathrm{moving}\;\mathrm{down}\right)\\ \left(\;-\#\mathrm{nonevents}\;\mathrm{moving}\;\mathrm{up}\right)/\#\mathrm{nonevents}) \end{array}$$

where events moving up or down are more or less likely, respectively, to experience events as rated by the new model. The NRI ranges from 0–2. Statistical analyses were performed using SAS 9.2 (Cary, NC).

## Results

### Patient characteristics

The contemporaneous echocardiography cohort subset was similar to the parent reproducibility cohort (Table 1). In the echocardiography cohort, the median time difference between echocardiography and CMR was 3 days (IQR 1–9 days). Most CMR exams were ordered to evaluate for scar in either the ischemic or nonischemic setting, or to evaluate for arrhythmogenic substrate; approximately 1 in 5 CMR scans employed vasodilator stress testing. In 355 subjects (80%), echocardiography preceded CMR. The final reports indicated that 76 of the 444 (17%) echocardiograms were rated as technically difficult by the interpreting physician.

**Table 1:** Patient characteristics

**Variable**	**Reproducibility cohort**	**Contemporaneous echocardiography cohort**
	**Frequency or median (interquartile range) N = 1044**	**Frequency or median (interquartile range) N = 444**
*Demographics*		
Age (years)	54 (42–65)	55 (44–65)
Female	41%	36%
White race	88%	88%
Black race	8.5%	8.1%
		
*General Indication for CMR exam*		
Known or suspected cardiomyopathy	31%	36%
Possible coronary disease (stress testing or viability)	31%	30%
Vasodilator stress testing	17%	17%
Evaluation for arrhythmia substrate	21%	25%
Mass or thrombus	3%	3%
		
*Hospitalization status*		
Inpatient	33%	36%
		
*Comorbidity*		
Hypertension	45%	51%
Diabetes	17%	19%
Dyslipidemia	35%	37%
Current cigarette smoking	15%	20%
Atrial fibrillation or flutter	6%	8%
Body mass index (kg/m^2^)	28.1 (24.4-33.4)	28.1 (24.1-33.2)
Obstructive coronary artery disease (>70% by angiography)	19%	20%
Prior coronary bypass	8%	8%
Prior percutaneous coronary intervention	14%	15%
Prior heart failure documented in medical records	18%	22%
*Laboratory, CMR, or Echocardiography data*		
Glomerular filtration rate (mL/min/1.73 m^2^)	82 (66–102)	81 (66–105)
Ejection fraction by echocardiography (%)	58 (34–63)	58 (38–63)
Ejection fraction by CMR (%)	58 (47–65)	55 (38–63)
Late gadolinium enhancement (LGE)	42%	52%
Myocardial infarction by LGE	19%	22%

### Reproducibility of CMR survival data

**Figure 1:**
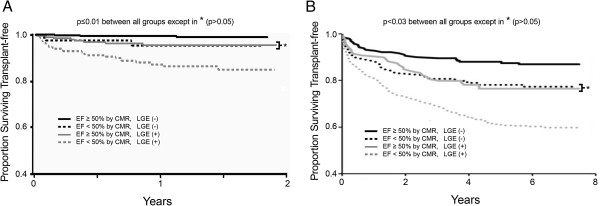
**Generalizability is important for newer imaging modalities, and our survival data of consecutive patients stratified by cardiovascular magnetic resonance (CMR) findings reproduced the results of others.** Our data (n = 1044) in panel **A** yielded similar relationships as initially described by Cheong et al., [5] reproduced in panel **B** (n = 857). Permission to reproduce the figure in panel **B** was granted by the publisher (Wolters Kluwer Health).

In the larger reproducibility cohort, there were 38 deaths and 3 cardiac transplants over a median follow-up of 1.0 years (IQR 0.4-1.5 years). Using CMR data only, we obtained very similar relationships between the Kaplan-Meier survival curve strata for transplant free survival compared to the prior work of Cheong et al. [5] as shown in Figure 1. Thus, simple risk stratification schemes based on EF < 50% and LGE were reproducible and generalized to our center.

### Ejection fraction by echocardiography or CMR and relation to mortality

**Figure 2:**
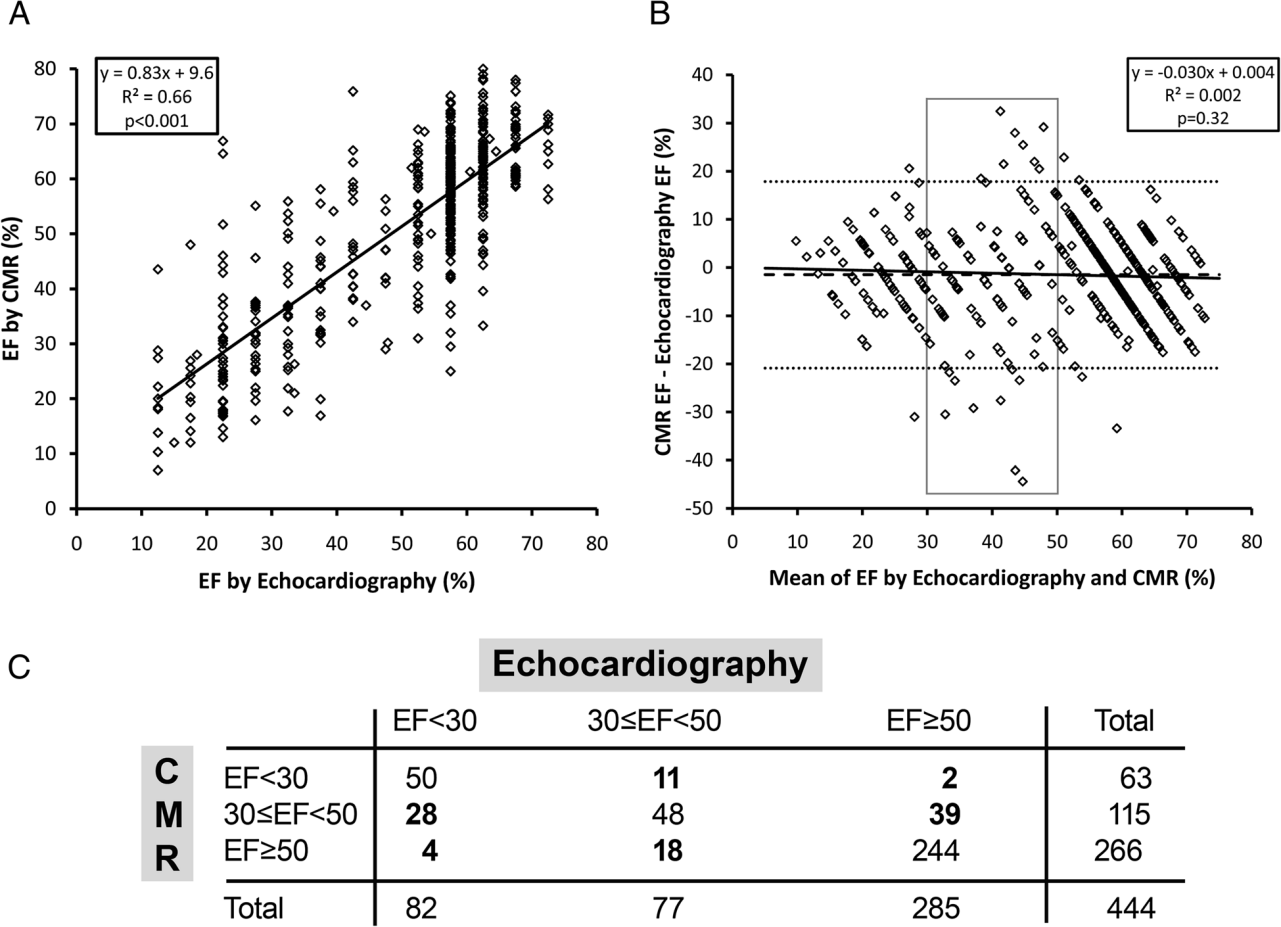
**Ejection fraction (EF) measured by contemporaneous echocardiography and cardiovascular magnetic resonance (CMR) correlate only moderately (panel A), and there is considerable scatter and misclassification.** Bland-Altman analysis (panel **B**) reveals that this scatter does not result from systematic bias. Importantly, most of scatter occurs in the 30%-50% range of the EF spectrum where clinical decision making relies most heavily on EF measures as shown by the thin gray box in panel **B**. Of note, variation far exceeded the ∆ 5% increment used for EF reporting by echocardiography. In Panel **C**, despite similar median EF values and the absence of meaningful bias in the EF measures of the *population*, the scatter exhibited by the *individual* differences in echocardiography and CMR EF measures culminate in 102 individuals (23%) of the sample being categorized differently (highlighted in bold font).

While EF by echocardiography and CMR were correlated and exhibited similar median EF measures (58% vs. 55%), the correlation plot exhibited considerable scatter (Figure 2), and CMR only explained 2/3 of the variation in EF by echocardiography as shown by the R^2^ of 0.66. The relation between CMR EF and echocardiography EF was not influenced by the time difference between the two tests (p = 0.8), and there was no interaction with time between the two tests (p = 0.7). The differences in EF did not vary as a function of the time difference between the two tests (p = 0.9). Bland Altman analysis revealed minimal bias (Figure 2). Importantly, disagreement was most prominent in the middle of the EF spectrum where clinical decision making relies most heavily on accurate EF determination. When the EF measures were placed into clinically meaningful categories (i.e., EF < 30% which indicates potential eligibility for defibrillator implantation, or EF <50% which indicates eligibility for medical therapy [16]) 102 individuals (23%) were classified differently (Figure 2, panel C).

There were 30 deaths and 3 cardiac transplants in 444 patients in the echocardiography cohort over a median follow-up of 0.83 years (IQR 0.29-1.40 years). In univariable Cox regression models, contemporaneous EF measures by echocardiography or CMR were both related to mortality (HR 1.05, 95% CI 1.02-1.07, and HR 1.05, 95% CI 1.03-1.07, respectively for every 1% decrement in EF). Yet, the association with all cause mortality or transplant free survival measured by the Wald *χ*^2^ was higher for EF measured by CMR compared to EF measured by echocardiography (21.5 versus 17.3 for death, respectively; 26.0 versus 20.1 for transplant free survival, respectively).

**Figure 3:**
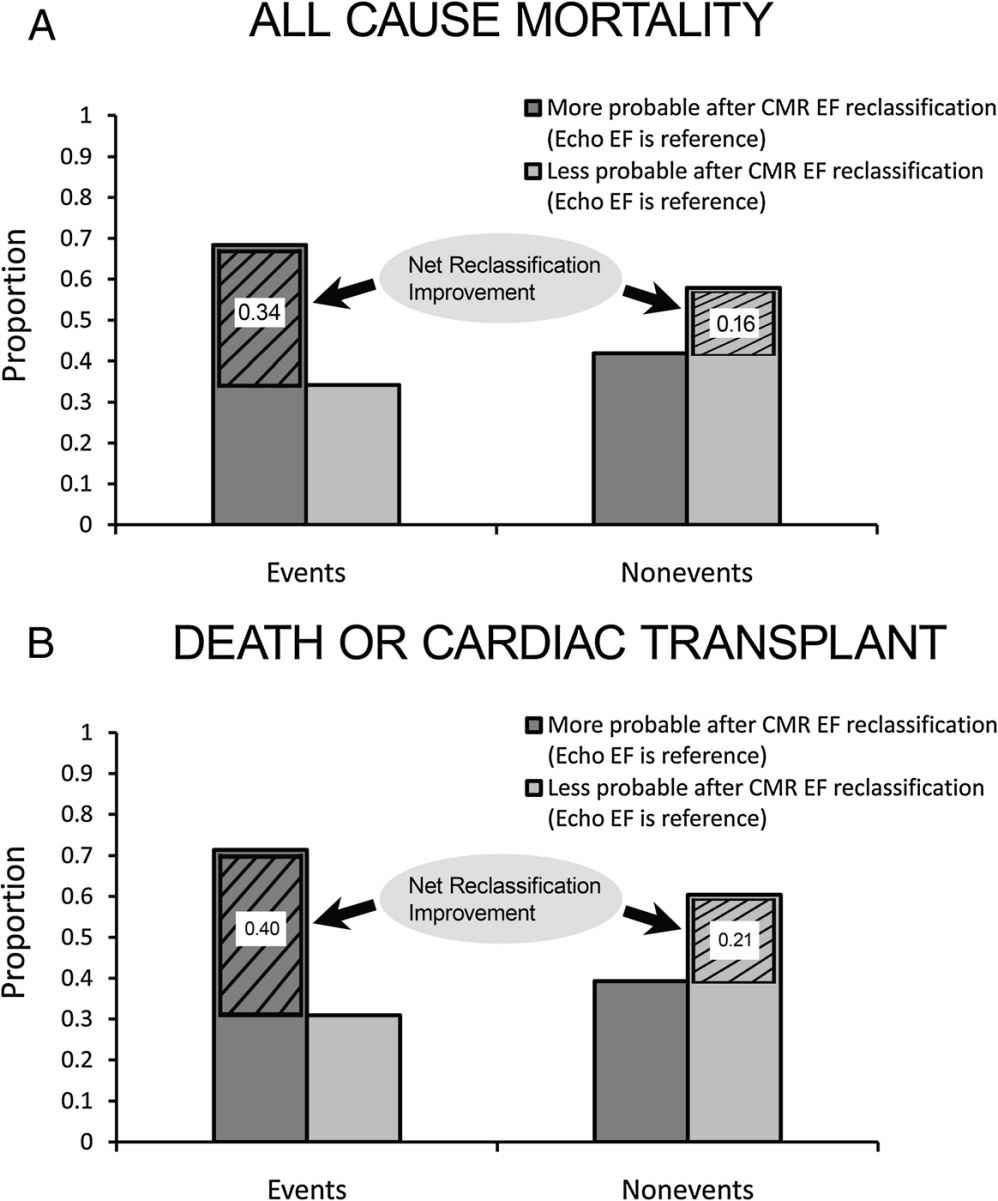
**Graphical depiction of the Net Reclassification Improvement (NRI) where univariable Cox regression models with cardiovascular magnetic resonance (CMR) ejection fraction (EF) predicting all cause mortality (panel A) or death or cardiac transplant (panel B) are compared to Cox regression models containing echocardiography (echo) EF.** Since the reclassification improved using CMR EF relative to echocardiography EF in *both* those with events and those without events, the NRI for all cause mortality and death/cardiac transplant are 0.51 (includes rounding error; panel **A**) and 0.61 (panel **B**), respectively, after summing these net improvements for events and nonevents.

A univariable Cox regression model with CMR EF significantly improved prediction of adverse events and significantly reclassified patients at risk compared to a univariable Cox regression model with echocardiography EF (mortality: IDI 0.018, 95% CI 0.008-0.034; NRI 0.51, 95% CI 0.11-0.85; transplant free survival: IDI 0.026, 95% CI 0.013-0.045; NRI 0.61, 95% CI 0.25-0.94). These data, shown graphically in Figure 3 indicate that CMR EF stratifies risks of adverse events significantly better than echocardiography EF, and that CMR EF reclassifies individual patients culminating in improved prediction of adverse events.

### Added prognostic value of late gadolinium enhancement

**Figure 4:**
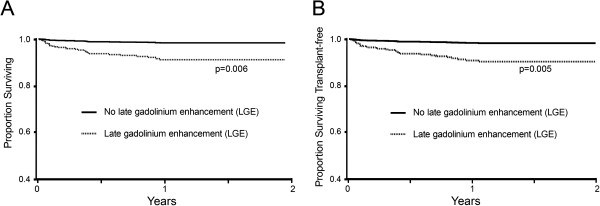
***Adjusted *Kaplan Meier survival curves for all cause mortality (panel A) and death or cardiac transplantation (panel B) according to late gadolinium enhancement (LGE) accounting for variation in age, ejection fraction by echocardiography, and regional wall motion abnormalities detected by echocardiography.**

In the echocardiography cohort, adding LGE data (expressed as a binary variable) to Cox models containing contemporaneous EF measured by echocardiography improved risk stratification. Adjusting for age, EF measured by echocardiography, and regional wall motion abnormalities detected by echocardiography, the presence of LGE was strongly associated with adverse outcomes (HR 5.44, 95% CI 1.62-18.3 for death; HR 5.71, 95% CI 1.71-19.1 for death or cardiac transplant) as shown in Figure 4. CMR data in the form of LGE alone refines risk stratification even with pre existing contemporaneous echocardiography EF.

LGE added also predictive ability beyond EF as determined by the Net Reclassification Index (NRI) and the Integrated Discrimination Improvement (IDI) in all multivariable Cox regression models predicting all cause mortality or predicting transplant free survival as shown in Table 2. These data indicate that LGE significantly improves the Cox regression model risk stratification for death or transplant free survival beyond age, gender, regional wall motion assessment by echocardiography, and EF measurement, regardless of whether EF was measured by CMR or echocardiography. Specifically, LGE improves the discrimination of the models (IDI) and improves the classification of individual patients (NRI).

**Table 2:** Late gadolinium enhancement (LGE) added significant predictive ability for either death or transplant free survival beyond ejection fraction (EF), age, regional wall motion by echocardiography and gender in Cox regression models regardless of how EF was measured

** Multivariable Cox regression model:**		**Adjusted hazard ratio for LGE (95% CI)**	**NRI (95% CI)**	**IDI (95% CI)**
**Outcome**	**EF modality**			
Mortality	CMR	3.84 (1.11-13.4)	0.61 (0.30-0.92)	0.017 (0.005, 0.028)
Mortality	echocardiography	4.44 (1.30-15.2)	0.72 (0.40-0.98)	0.024 (0.008, 0.040)
Transplant free survival	CMR	4.02 (1.16-13.9)	0.64 (0.35-0.93)	0.018 (0.005, 0.030)
Transplant free survival	echocardiography	4.79 (1.41-16.3)	0.74 (0.46-0.99)	0.027 (0.009, 0.044)

All models stratified by gender and adjusted for age, regional wall motion abnormalities by echocardiography and ejection fraction (EF) by either echocardiography or CMR. The net reclassification improvement (NRI) and integrated discrimination improvement (IDI) reflect the added performance of the model after LGE data are added.

## Discussion

This study supports the utility of CMR after contemporaneous echocardiography in selected patients. We demonstrate for the first time that CMR can refine prediction of mortality and transplant free survival, even when contemporaneous 2D echocardiography with EF and wall motion assessment is available. Our data suggest that CMR derived EF and 2D echocardiography derived EF assessed visually are not always equivalent for individual patients, not because of systematic bias, but because of imprecision by echocardiography. Among those referred for CMR, echocardiography derived EF appeared to misclassify 23% of patients when EF is divided into clinically relevant categories. Accordingly, CMR derived EF was a significantly better predictor of all cause mortality or transplant free survival in our cohort. Furthermore, beyond issues related to EF measurement, LGE data in particular may significantly improve the prediction of all cause mortality and transplant free survival beyond preexisting echocardiography EF data and wall motion assessment. Thus, even when echocardiography data already exist, CMR data in selected patients can provide more robust EF assessment and additional information in the form of LGE that further improves risk stratification beyond EF. Finally, we also reproduced the relationships between EF/LGE survival curve strata previously reported by others, [5] affirming the apparent generalizability of CMR EF and LGE data.

Whether our findings extend to those not referred for CMR is uncertain and beyond the scope of our study. Nonetheless, our data suggest the potential for improved risk stratification with CMR EF and LGE data in selected patients. Indeed, CMR has been proposed as a clinically robust technique to assess myocardial tissue and regional and global function. LGE detection of key myocardial changes such as necrosis or fibrosis has been previously validated [17-19] and has been associated with improved diagnosis of prognostically relevant, clinically unrecognized myocardial infarction, [20-23] adverse outcomes, [5,20-24] and limited functional recovery after revascularization [25] or medical therapy. [26] Similarly, EF by CMR also has been validated. [27] The precision of EF estimates by 2D echocardiography may be improved by more liberal use of echocardiography contrast agents [28] or the modified Simpson’s rule. [2] Still, CMR provides 3D *volumetric* assessment for EF measurement which is superior to 2D biplane techniques and is more reproducible. [1,8] An important observation from our study and others [28] was that the discrepancies in EF measurement were largest in the intermediate range as shown in the Bland-Altman analysis from Figure 1. The intermediate range of EF (e.g., 30-50%) is where clinical decision making is more nuanced and possibly more reliant on accurate and precise EF measurement.

Conceptually, we believe our data can support a clinician’s decision to refer patients to CMR. When clinicians chose to refer patients for CMR, it generally yielded incremental prognostic information in our cohort. Still, it is doubtful that CMR can ever accommodate the volume of echocardiography laboratories which incur less cost. Therefore, the challenge to the cardiology community is how best to utilize CMR and echocardiography. More data are needed to identify 1) whether the improved risk stratification associated with CMR might improve patient centered outcomes and cost effectiveness relative to echocardiography-directed care through improved matching of patients and interventions, and 2) whether CMR may permit greater efficiency in the allocation of limited healthcare resources. For example, CMR may have value as a first line modality for those with known decrements in EF, or susceptibility to poor acoustic windows such as the obese or those with lung disease. Preliminary knowledge from our study that CMR with LGE has the potential to improve the classification of individual patients at risk provides an important foundation for justifying the significant costs and efforts associated with implementing such research. Even if echocardiography yielded identical EF measures as CMR, possibly with 3D techniques, LGE would still provide added prognostic value as shown in Table 2. Novel CMR techniques to quantify the extracellular volume fraction might refine risk stratification even further [29-35].

Ultimately, these data may have implications for clinical practice, for trials requiring risk assessment, and for efforts to gauge comparative effectiveness and improve processes of care. We speculate that for selected patients, the improvements in risk stratification offered by CMR (quantified in Table 2) may justify the higher associated upfront costs and more complex infrastructure needed to support CMR practice. Conceptually, CMR in selected patients could reduce downstream costs and minimize adverse events through improved selection of truly high risk patients for costly (relative to CMR) and invasive procedures that also carry inherent risk (e.g., defibrillator implantation). Ideally, overuse of expensive and invasive procedures could be minimized by identifying truly low risk individuals unlikely to benefit who can avoid procedure-related risks, and underuse can be minimized by identifying truly high risk individuals likely to benefit despite the procedure-related risks. Further study is undoubtedly needed to test these hypotheses which remain unconfirmed.

### Limitations

Our study has limitations. First, as with all observational studies, our sample has inherent selection/referral biases so our results may not generalize to those *not* referred for CMR. Nonetheless, we note that LGE is unique to CMR, and no echocardiography report in our study suggested sufficient uncertainty in EF determination to warrant further EF determination by alternative imaging modalities. Second, our results are obtained from a single center with a dedicated CMR scanner and significant underlying infrastructure committed to supporting CMR, so results may not generalize to centers without CMR expertise. Still, the availability of CMR is growing, and we note that we could reproduce the CMR results of others who demonstrated previously that LGE improves risk stratification, suggesting that our center’s experience may not necessarily be unique. Finally, EF by echocardiography was mostly estimated visually which may represent a limitation. Yet, qualitative assessment (often without echocardiography contrast) is widespread, affirming the relevance of our data, and American Society of Echocardiography (ASE) Guidelines [2] do not specify optimal 2D echocardiography measurement techniques for EF, i.e., qualitative versus modified biplane Simpson’s method. While use of Simpson’s method or 3D echocardiography may yield different results, ASE guidelines still recommend cross checking quantitative attempts with qualitative estimates, and others have reported that Simpson’s method has inherent limitations compared to 3D tomographic techniques such as CMR [1-3,8].

## Conclusions

In selected patients referred for CMR in a real world setting, CMR with LGE may improve risk stratification for death or the combined endpoint of death/cardiac transplantation after contemporaneous echocardiography. LGE appears to provide additional prognostic ability beyond ejection fraction. Also, visual EF measures by echocardiography and CMR are not always equivalent. Individuals may be classified differently by CMR after echocardiography, and EF measurement by CMR may better predict subsequent mortality or transplant free survival compared to EF measurement by echocardiography. The similarity of the relationships between EF and LGE to that reported by others [5] suggests our CMR data are generalizable. These data may have implications for efforts to optimize care, assess effectiveness, and allocate limited health care resources. We hope this study will promote allocation of additional resources needed to explore these issues further.

## Abbreviations

CMR: Cardiovascular magnetic resonance; LGE: Late gadolinium enhancement; IDI: Integrated discrimination improvement; NRI: Net reclassification improvement; 3D: 3 dimensional; 2D: 2 dimensional; EF: Ejection fraction.

## Competing interests

Dr. Schelbert has served as an unpaid scientific advisor to Siemens Medical Solutions. The remaining authors declare that they have no competing interests.

## Authors’ contributions

TCW and EBS conceived of the study design and drafted the manuscript. PK optimized CMR acquisition protocols. KP, KSP, DM, CGM, TCW and EBS collected the data. EBS performed the statistical analysis. TCW, JML and EBS interpreted CMR scans. All authors provided critical revision of the manuscript. All authors read and approved the final manuscript.
